# Assessment of the Ecological Compensation Standards for Cross-Basin Water Diversion Projects from the Perspective of Main Headwater and Receiver Areas

**DOI:** 10.3390/ijerph20010717

**Published:** 2022-12-30

**Authors:** Yubing Wang, Kai Zhu, Xiao Xiong, Jianuo Yin, Haoran Yan, Yuan Zhang, Hai Liu

**Affiliations:** 1 Hubei Key Laboratory of Regional Development and Environmental Response, Hubei University, Wuhan 430062, China; 2 Research Center of Territorial Space Management, Hubei University, Wuhan 430062, China; 3 Faculty of Resources and Environmental Science, Hubei University, Wuhan 430062, China; 4 Ecological Environment Monitoring Center of Guizhou Province, Guiyang 550081, China; 5 Ecological Environment Monitoring Center Station of Hubei Province, Wuhan 430071, China

**Keywords:** payment for ecosystem services, ecological compensation standard, cross-basin water diversion projects, south-to-north water diversion project

## Abstract

This paper aims to explore how to develop reasonable ecological compensation standards to improve the effectiveness of water diversion projects. Watershed ecological compensation is an important means to coordinate watershed protection and development and, additionally, compensation standard accounting is the core issue of ecological compensation. The previous literature has mainly calculated watershed ecological compensation standards from a single perspective, such as the main headwater or receiver areas, meaning the interests of another under-appreciated area would inevitably be ignored. The calculation results of different perspectives and methods vary greatly, directly affecting the implementation of watershed ecological compensation mechanisms. In this paper, the world’s largest water diversion project, the Middle Route of the South-to-North Water Diversion Project, was selected as the study area. The total cost correction model was selected from the perspective of the main headwater areas. The water resources input-output model was selected from the perspective of the receiver areas to evaluate the ecological compensation criteria and compare the differences between the two models. The results show that the ecological compensation standards based on the perspective of water source areas are mainly influenced by the ecological construction expenditures and industrial opportunity cost losses in the watershed, with higher compensation costs in the early period but a more moderate growth trend in the later period. The ecological compensation standards based on the perspective of the receiver areas increase with the annual increase in project water diversion, with a low compensation cost in the early period, but a faster growth trend in the later period. The ecological compensation standards calculated by different perspectives and methods differ significantly; the main contribution of this paper is to enrich the ecological compensation research on cross-basin water diversion projects from multiple perspectives.

## 1. Introduction

Water resources play a vital role in human survival and development as they constitute an important part of natural resources [1]. As society and the economy grew rapidly, the overexploitation of water resources has led to increasing damage to the watershed ecological environment. The natural environment that human beings depend on is under serious threat [2]. Ecological compensation has gradually developed into an important means to protect the ecological environment around the world and is a hot topic studied by scholars, for it provides a new way to solve cross-basin ecological problems [3]. Ecological compensation is a method of commercializing natural ecosystem services and is widely used in the fields of environmental management and natural resources management. Ecological compensation works by building a well-structured natural resources trading market, determining the specific price of natural resources according to the actual needs of buyers and sellers and encouraging landowners to trade idle resources in the market in a sustainable manner, thus effectively reducing people’s harmful externality activities [4]. When applied to watershed fields, it is called watershed ecological compensation [5,6]. Watershed ecological compensation should follow the principle of “who develops, protects; who destroys, restores; who benefits, compensates; who pollutes, pays” and should aim to strengthen the paid use of water resources and the “polluter pays” policy through economic means [7]. The key advantage of watershed ecological compensation is that it can coordinate different administrative regions to solve cross-basin ecological problems through economic means instead of focusing only on pollution problems in its own jurisdiction [8]. Therefore, watershed ecological compensation has been widely recognized by the international community and has been widely implemented in many countries and regions [9].

Reasonable compensation standards are the core of watershed ecological compensation and the key to motivating ecosystem service providers and achieving regional synergistic development [10]. In general, watershed ecological compensation includes both rewards for benefits gained from protecting watershed ecosystems and water resources, or compensation for losses caused by their destruction, as well as fees for those who cause environmental pollution in the watershed [4,11]. For ordinary watersheds, watershed ecological compensation refers to the compensation for damage caused by environmental pollution downstream because of upstream discharges exceeding the purification capacity of the watershed, as well as the loss of the cost of environmental protection measures such as soil and water protection that the upstream chooses to adopt at the expense of its regional self-development opportunities, resulting in no pollution downstream [12]. Therefore, the watershed ecological compensation standard is the basic measure of the loss of the compensated subject and the benefit of the paying subject.

The construction of cross-basin water diversion projects has realized the rational allocation of water resources and solved the water shortage problem in large areas [13,14]. Many countries have implemented various cross-basin water diversion projects to bring a large amount of clean water resources to the receiver areas and to create high economic benefits. At the same time, the main headwater areas of water diversion projects pay a considerable price to ensure a long-term stable supply of clean water resources. Therefore, it is imperative to establish an ecological compensation mechanism for cross-basin water diversion projects, considering the ecological protection and coordinated socioeconomic development of the main headwater areas [15].

Through watershed ecological compensation, the separation of interests between the receiver and headwater areas of water diversion projects and the asymmetry between investment and revenue can be effectively mitigated [16,17]. Policy support and watershed ecological compensation for the main headwater areas would optimize the industrial and economic structures, leading its ecological construction and economic development to go hand in hand, improving the living standard of its residents, and encouraging the behavior of protecting the watershed ecological environment. The improvement of the ecological environment of the water source areas for the receiver areas causes them to be capable of providing better and more sustainable ecological service products and deepens their awareness of the ecological value of water transfer through their gained benefits, which results in paying more attention to the compensation behavior and consequently forming a virtuous circle to achieve synergistic development between the main headwater and receiver areas. Overall, the key advantage of watershed ecological compensation lies in coordinating the interests of different governments through transfer payments to achieve synergistic management of the water environment, to solve cross-basin water pollution issues, and ultimately to achieve the sustainable use of water resources [18,19].

At present, an increasing number of scholars are devoted to watershed ecological compensation research and have attained a certain level of achievement in the fields of compensation theory, modeling, and standard setting [20,21,22]. However, there are still various problems with watershed ecological compensation in practice, the most important of which is that it is difficult to formulate compensation standards accurately, leading to differences in the amount, form, and commutation of ecological compensation, which results in the implementation of watershed ecological compensation to be lacking in fairness and objectivity and seriously hinders the sustainable development of the benefits of water diversion projects [23,24,25,26].

At present, the international mainstream ecological compensation standard accounting methods mainly include willingness-to-pay, total cost accounting, and ecosystem services value methods [24], but most of the literature has adopted a single perspective and thereby only accounted for the ecological compensation standard in the main headwater or receiver areas. In contrast, few papers have compared the differences in ecological compensation standard accounting methods and results by examining different perspectives. Therefore, it is necessary to study the ecological compensation standard of cross-basin water diversion projects from multiple perspectives. It is significantly important to establish a scientific and reasonable watershed ecological compensation mechanism.

As the world’s largest and most invested cross-basin water diversion project, China’s South-to-North Water Diversion Project (SNWDP) has been planned around three water transfer routes (the eastern, middle, and western routes) to transfer water resources from the Yangtze River Basin to the Huaihe River Basin and Haihe River Basin [27,28,29] and it has become the primary source of water for domestic use in several important cities [15]. The water diversion project has brought high economic benefits to the receiver areas [30]. However, while the receiver areas enjoy the clean water and high economic benefits brought by the water diversion project, the main headwater areas do not benefit from it and they bear high ecological protection costs of the watershed [29]. The upstream Hanjiang River Basin (HJRB) in the water diversion district of the Middle Route Project (MRP) is an economically underdeveloped area and many of its economic indicators are lower than the national level. To ensure that the transferred water quality and quantity from the source area meet the requirements of the water diversion project, it is necessary to invest a large amount of money to carry out environmental protection and pollution prevention work in the basin and to sacrifice certain local economic development opportunities, i.e., opportunity costs [16]. In contrast to the high investment in ecological protection and opportunity costs, the state financial subsidies appear to be minimal; therefore, it is necessary to provide more support to the water diversion areas through watershed ecological compensation to achieve a win-win situation in terms of high-quality water resource protection and socioeconomic development of the main headwater areas.

This paper focuses on the application of different compensation standard calculation methods in the MRP of SNWDP from the perspectives of the main headwater and receiver areas. The main research questions of this paper are as follows:

- Based on the perspective of main headwater areas, the traditional total cost model is modified by introducing the water quantity allocation and water quality correction coefficients from the ecological protection cost and opportunity cost paid by the main headwater areas to provide high-quality water resources.
- Based on the perspective of the receiver areas, a new water resources input-output model is constructed from the generated economic benefits by using the diverted water resources in the receiver areas and combining the regional input-output table and cost analysis method.
- In this paper, the total cost correction model and the water resources input-output model are used to calculate the watershed ecological compensation standard of the world’s largest cross-basin water diversion project and to compare the differences between the two methods.

The main aim of this study was to evaluate the ecological compensation standards of cross-basin water diversion projects from the perspectives of main headwater and receiver areas, respectively, and to compare the differences in the results of different perspectives and methods to further explore how to develop reasonable ecological compensation standards to improve the effectiveness of water diversion projects. The structure of this study is arranged as follows: after introducing the case areas (Section 2), theory (Section 3), and methodology (Section 4), ecological compensation criteria from different perspectives were discussed (Section 5), their differences, theoretical contributions, policy measures, and future work were compared (Section 6), and then the final conclusion of this paper was provided (Section 7).

## 2. Case Overview

The SNWDP is currently the world’s largest cross-basin water diversion project [28,30,31]. The project is a major strategic infrastructure to alleviate water shortages and improve the ecological environment in northern China [15,32]. In December 2014, after 11 years of construction, the MRP was officially opened with a total length of 1432 km. The project focuses on solving water shortages in four provinces and cities in Henan, Hebei, Beijing, and Tianjin and provides water for cities and regions along the project route. In addition, the function of the MRP is to improve the urban water and ecological environment and indirectly to supply water for agriculture to an area of 155,000 square kilometers within its water supply area [33].

**Figure 1:**
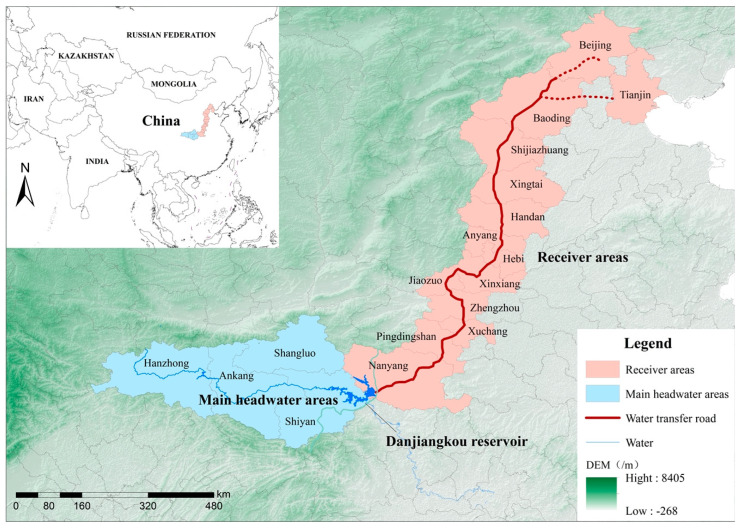
The location of the target study area.

The MRP can be divided into three main parts: the main headwater areas, water delivery channel, and receiver areas. Among them, the main headwater areas are the upstream area of the HJRB, mainly including Hanzhong, Ankang, Shangluo, and Shiyan. The channel spans the Henan, Hebei, Beijing, and Tianjin administrative regions. The receiver areas include 14 large- and medium-sized cities such as Nanyang, Pingdingshan, Xuchang, Zhengzhou, Jiaozuo, Xinxiang, Hebi, Anyang, Handan, Xingtai, Shijiazhuang, Baoding, Beijing, and Tianjin [29]. Figure 1 shows the MRP’s main headwater areas, receiver areas, and transfer routes.

Between 2011 and 2015, to guarantee the delivery of high-quality water resources to the receiver areas, the main headwater areas shut down more than 500 seriously polluting enterprises, banned more than 1000 “ten small” enterprises, stopped more than 300 new projects, managed soil erosion over 20,000 square kilometers, and maintained the overall water quality of the water source at Class II [30]. The MRP has alleviated the water shortage problem in central and northern China, significantly improved the ecological environment and investment environment in the receiver areas, and strongly promoted economic and social development. The MRP was officially opened in December 2014 and, as of July 2022, the MRP has transferred a cumulative total of more than 50 billion cubic meters of water, benefiting more than 85 million people [34].

## 3. Theoretical Background

As an independent natural geographical unit with high correlation, complexity, and a multilevel nature with other natural units [25,35,36,37], the watershed is a popular research object in ecological compensation [16,22,38]. The watershed ecological compensation standard accounting method has gradually become a research hotspot [39]. Previously, research on the watershed ecological compensation standard accounting method was mainly a one-way compensation, i.e., the beneficiary compensates the service provider [11]. Nevertheless, recently, more scholars have been focusing on two-way compensation, i.e., connecting beneficiaries and service providers in a model of incentives and compensation [4].

At present, the international mainstream ecological compensation standard accounting methods mainly include willingness-to-pay [40], total cost accounting [41], ecosystem service value [24,42], water resources value [20,43], and water quality and quantity protection target accounting methods [44,45,46]. The quantification of watershed ecological compensation standards does not have a unified system. However, in regard to the issue of watershed water resource trading, the trading price and willingness to pay are always difficult to unify [7]. Therefore, studying watershed ecological compensation standard accounting methods can provide a theoretical basis for solving the contradiction in watershed development.

There are many methods of watershed ecological compensation standard accounting and different methods are used to arrive at different compensation costs; even their calculation results have significant differences. The advantages and disadvantages of the various compensation standard accounting methods are shown in Table 1. The willingness-to-pay method is widely used and is considered a mature method in the international arena. However, because of the special nature of the society and economic model in China, this method is not easy to promote [47]. Yisheng Ren conducted a face-to-face questionnaire-based survey of 2217 randomly selected local residents and concluded that the crucial factors affecting willingness to pay ecological compensation for the Xin’an River were government employees, individual households, and government staff as family members [48]. The beneficiaries of watershed ecological services easily accept the total cost accounting method. However, the accounting criteria have not been scientifically certified, especially for calculating indirect costs, which in practice are usually less than their value [3]. Lina Sun used a semi-distributed hydrological SWAT model to establish a compensation standard considering land use changes with the combined application of remote sensing and geographic information systems, arguing that the operationalization and accuracy of cost–benefit analysis is difficult and controversial [49]. The ecosystem service value method is currently used to simulate changes in the value of ecosystem services and translate them into monetary values through the InVEST model, but the results of this method are often unacceptable to beneficiaries and have low public acceptance [21,50]. Xin Gao used the ecosystem services value method to calculate the ecosystem services value of Jiangsu Province, then the change of this ecosystem services value in the Jiangsu Province is used as the basis for watershed ecological compensation standards. However, the authors also consider this result controversial [24]. Junfei Chen tries to establish the payment standard of watershed ecological compensation from the perspective of water resources value [20]. Nevertheless, the water resources value method is extremely dependent on market regulation and can only be effectively used when the water resources market is stabilized [20,51]. The water quality and quantity protection target accounting method is a widely used accounting standard in China’s watershed ecological compensation practice and it has a two-way incentive effect, meaning it enables beneficiaries and service providers to work together on water conservation in a better manner [46]. However, practice shows that this approach only applies to situations where the level of regional economic development is the same in the considered areas [44].

All commonly used watershed ecological compensation standard accounting methods have their own limitations. To compensate for their shortcomings, this paper explores the combined use of various accounting methods to complement each other and to establish a scientifically based ecological compensation accounting system for the natural geography cross-basin water diversion projects, taking into consideration their social and economic development.

Cross-basin water diversion projects include the main headwater areas (service provider) and the receiver areas (beneficiary). Based on the service provider’s perspective, the total cost accounting method can be combined with the water quality and quantity protection target accounting method. The modified total cost method not only considers the cost paid by the service provider but also focuses on the benefits of the water diversion project. Based on the beneficiary perspective, the water resources value, the input-output and the willingness-to-pay method can be combined and the ecological compensation standard can be assessed from the perspective of the economic benefits obtained by the use of the transferred water resources in the receiver areas.

## 4. Data and Methods

### 4.1. Data

The data used in this paper come from the 2015–2020 “Statistical Development Bulletin”, “Soil and Water Conservation Bulletin”, “Statistical Yearbook”, “China South-North Water Diversion Project Construction Yearbook”, and water quality monitoring data of key cross-sections from the China National Environmental Monitoring Centre of each province and city in the main headwater and receiver areas.

### 4.2. Methods

#### 4.2.1. Total Cost Correction Model

The fundamental purpose of the main headwater areas in terms of supporting ecological construction projects is to provide quality water for the receiver areas. For this reason, implementing ecological and environmental construction projects requires large investments. Moreover, the main headwater areas lose many development opportunities due to development restrictions [52]. Based on this consideration, the ecological compensation standard for the main headwater area protection can be measured in terms of both direct cost and opportunity cost; the calculation formula is as follows:

(1) $$C_{st}=C_{d}+C_{o}$$

where $C_{st}$ denotes the total cost of watershed ecological protection, $C_{d}$ denotes the direct cost of watershed ecological protection, and $C_{o}$ denotes the indirect cost of watershed ecological protection.

This method is an alternative method to calculate the positive environmental externalities. Its advantage is that it visually represents the financial and resource status of the main headwater areas in terms of protecting the environment and overcoming the deficiency created by the ecological service value being too high due to the lack of market basis and thereby deviating from the actual requirements of compensation [24,53].

##### Direct Cost

Direct cost refers to the main headwater areas for protecting and constructing the water source ecological environment and the direct investment in human, material, and financial resources [54]. Currently, the scope and standard of direct cost accounting have not yet been formed as a unified and clear index system. According to the previous literature and considering it with the actual situation of the study area, the scope of direct cost accounting is determined in this paper as two categories: ecological environmental protection and construction costs and integrated pollutant management costs [55,56]. Among them, ecological environmental protection and construction costs include forestry construction costs, soil and water conservation costs, and watershed ecological construction costs. Integrated pollutant management costs include hydrological monitoring costs and water pollution control costs. The calculation formula is as follows:

(2) $$C_{d}=C_{epc}+C_{ipm}$$

where $C_{d}$ denotes the direct cost of watershed ecological protection, $C_{epc}$ denotes the cost of ecological environmental protection and construction, $C_{ipm}$ denotes the cost of comprehensive pollutant management, and the interpretation of indicators is shown in Table 2.

##### Opportunity Cost

The scarcity of a resource causes opportunity costs. It refers to the potential loss of value resulting from another aspect of functional use that must be foregone in developing and utilizing a resource [54]. In watershed ecological compensation, the opportunity cost is generally the economic loss caused by the partial development rights that the main headwater areas are forced to forego for the sake of the basin-wide ecology [45]. In general, opportunity costs cannot be obtained directly through market pricing and indirect market pricing needs to be applied to estimate the opportunity cost loss of the corresponding subject.

Implementing ecological protection in main headwater areas involves opportunity costs [20,24]. For example, the implementation of projects such as returning farmland to forests and building various types of public welfare forests will inevitably cause changes in land use, i.e., the area planted with cash crops will be reduced accordingly and the income from agricultural industries will drop accordingly. In addition, the main headwater areas will introduce stricter environmental protection policies and implement more stringent enterprise access systems and standards to improve environmental protection, which will greatly limit the survival and development of some high energy-consuming and high-polluting enterprises. The opportunity cost loss caused by ecological conservation behavior is mainly generated in the primary and secondary industries and the impact on the tertiary industry is slight. Therefore, this paper determines the scope of opportunity cost accounting as the opportunity cost of secondary industry producers and primary industry producers. The calculation formula is as follows:

(3) $$C_{o}=C_{si}+C_{pi}$$

where $C_{o}$ denotes the opportunity cost of watershed ecological protection, $C_{si}$ denotes the opportunity cost of secondary industry producers, and $C_{pi}$ denotes the opportunity cost of primary industry producers.

The opportunity cost of secondary industry producers is estimated by the growth rate of the secondary industry in the control areas and in the main headwater areas before and after the water diversion project. The calculation formula is as follows:

(4) $$C_{si}=GDP_{nl}\times N_{n}\times S_{n}$$

where *n* denotes the year of water transfer project implementation, $GDP_{nl}$ denotes the value of incremental loss of secondary industry per capita in year *n* after the start of the water diversion project, $N_{n}$ denotes the total population in year *n*, and $S_{n}$ denotes the revenue adjustment coefficient, which is the proportion of fiscal revenue in year *n* after the start of the water diversion project.

(5) $$GDP_{nl\mathrm{oss}}=GDP_{n-1}\times (1+a_{i}+\theta )-GDP_{n}$$

where $GDP_{n-1}$ and $GDP_{n}$ are the secondary industry output value per capita in years n−1 and year *n*, respectively, $a_{i}$ denotes the growth rate of the secondary industry value added in year *i* after the start of the water diversion project, and θ denotes the opportunity cost loss parameter.

The formula for the opportunity cost loss parameter θ is as follows:

(6) $$\theta =\left|\left(\alpha -\beta \right)-\left(\alpha^{'}-\beta^{'}\right)\right|$$

where α and $\alpha^{'}$ denote the average annual growth rate of the secondary value added per capita before and after the start of the water diversion project, respectively. β and $\beta^{'}$ denote the average annual growth rate of the secondary value added per capita in the control areas in the corresponding year.

The opportunity cost of producers in the secondary industry can be divided into two major categories: plantation and nonplantation. The non-planting industry can be subdivided into three sub-indicators: forestry, animal husbandry, and fishery. The calculation formula is as follows:

(7) $$C_{pi}=L_{1}+L_{2}+L_{3}+L_{4}$$

where $L_{1}$, $L_{2}$, $L_{3}$, and $L_{4}$ denote the producer opportunity costs of the plantation, forestry, livestock, and fisheries, respectively.

The plantation industry only needs to account for crop yield reductions due to changes in land use, so the plantation industry opportunity cost losses are calculated as follows:

(8) $$L_{1}=\sum_{i=1}^{n}P_{t1}\times S_{i1}$$

where $P_{t1}$ denotes the average price per unit area of a crop and $S_{i1}$ denotes the area planted for a crop land use change.

Forestry needs to consider the loss of economic benefits caused by the conversion of economic forests to public welfare forests, so the formula for calculating the opportunity cost loss in forestry is as follows:

(9) $$L_{2}=\sum_{i=1}^{n}P_{t2}\times S_{i2}$$

where $P_{t2}$ denotes the average return per unit area of a certain economic forest and $S_{i2}$ denotes the area of economic forest to public welfare forest.

The livestock industry can be calculated from the changes in the production of various types of livestock products corresponding to the loss of economic returns, so the formula for calculating the opportunity cost of the livestock industry is as follows:

(10) $$L_{3}=\sum_{i=1}^{n}P_{t3}\times \Delta R_{i3}$$

where $P_{t3}$ denotes the unit price of each livestock product and $\Delta R_{i3}$ denotes the reduced quantity of livestock products.

The fishery can be calculated by removing the area of net farming, so the formula for calculating the opportunity cost loss of the fishery is as follows:

(11) $$L_{4}=\sum_{i=1}^{n}P_{t4}\times S_{i4}$$

where $P_{t4}$ denotes the average return per unit area of a certain kind of net tank fish culture and $S_{i4}$ denotes the area of net tank removal.

##### Introduce Water Quantity and Water Quality Coefficient to Correct Total Cost

The total cost model sums up the main headwater areas’ direct input and opportunity costs and then introduces water quantity allocation and water quality correction coefficients to appropriately correct the calculated total costs and measure the final total cost amount [3]. The water quantity allocation coefficient is determined by the water diversion project water supply and the total water supply of the whole basin; the calculation formula is as follows:

(12) $$K_{Vt}=1+\frac{W_{MR}}{W_{HJRB}}$$

where $K_{Vt}$ denotes the water sharing coefficient and $W_{MR}$ and $W_{HJRB}$ denote the amount of water transferred and the total water supply of the whole basin, respectively.

In the process of cross-basin water transfer, the quality of water also affects the effectiveness of water resource use, so we further introduce the water quality correction coefficient through the water quality condition of the total cost of ecological protection for further adjustment [57,58,59]. The calculation formula is as follows:

(13) $$K_{Qt}=1+\sum_{t=1}^{n}\frac{R_{t}\times N_{t}}{C_{st}\times K_{Vt}}$$

where $K_{Qt}$ denotes the water quality correction factor, $R_{t}$ denotes the corresponding standard in the emissions of a pollutant, and $N_{t}$ denotes the reduction in unit emissions investment.

In summary, the total cost calculation formula after the introduction of the water quantity allocation and water quality correction coefficient is as follows:

(14) $$C_{real}=C_{st}\times K_{Vt}\times K_{Qt}$$

where $C_{real}$ denotes the modified total cost. The total cost correction model comprehensively covers the ecological protection construction cost. It is corrected by water quality and water quantity coefficients, which can more scientifically reflect the cost paid by the main headwater areas to maintain a good water source environment.

#### 4.2.2. Water Resources Input-Output Model

##### Input-Output Model

The receiver areas use the diversion water to create high economic benefits. Calculating the economic benefits of water diversion is the premise of studying ecological compensation standards [60]. Based on the water resources input-output model, a water resources shadow price calculation model is established to analyze the economic benefits generated by water diversion in the receiver areas by solving for the water resources shadow price [61,62].

The input-output model is a linear model reflecting the interdependence and constraint relationship between various industries in the national economy, the core of which is the input-output table, from which coefficients such as direct consumption and complete consumption can be obtained and, finally, the coefficients and variables to establish a function model according to the equilibrium relationship between the ranks in the input-output table can be combined [5,63]. The direct consumption factor is the amount of product of sector *i* that needs to be consumed directly to produce the total output of sector *j*. The formula is as follows:

(15) $$a_{ij}=\frac{z_{ij}}{x_{j}},\left(i,j=1,2\cdots ,n\right)$$

where $a_{ij}$ denotes the direct consumption coefficient, $z_{ij}$ denotes the amount of output of sector *i* consumed in the production of sector *j*, $x_{j}$ denotes the total input of sector *j*, and *n* denotes the number of sectors.

The complete consumption coefficient $b_{ij}$ denotes the sum of the direct and indirect consumption of the final product of production unit *j* in sector *i*. Matrices *A* and *B* are commonly used for input-output analysis to represent the whole direct and complete consumption coefficients.

(16) $$A=\begin{array}{ccc} a_{11} & \cdots & a_{1n}\\ \vdots & \ddots & \vdots \\ a_{n1} & \cdots & a_{nn} \end{array},B=\left[\begin{array}{ccc} b_{11} & \cdots & b_{1n}\\ \vdots & \ddots & \vdots \\ b_{n1} & \cdots & b_{nn} \end{array}\right]$$

The relationship between matrices *A* and *B* can be expressed as follows:

(17) $$B={\left(I-A\right)}^{-1}-I$$

where ${\left(I-A\right)}^{-1}$ is the Leontief inverse matrix and *I* denotes the unit matrix.

The input-output table is the most important analytical tool of the input-output model; the rows represent the output destination of all the sectors and the columns represent the input sources of all the sectors [64]. Water resources play an important role in all aspects of production and consumption in the national economy. Therefore, it is feasible to introduce the water consumption of each industry into the input-output table to form a water resources input-output table, based on which the input-output analysis of water resources can be carried out. Based on this, the water resources input-output model is constructed as follows:

(18) $$X={\left(I-A\right)}^{-1}Y$$

where *X* is the total output matrix, *Y* is the end-use matrix, and *A* is the direct consumption factor matrix.

The direct water use coefficient w for sector *j* is introduced as the ratio of water use in sector *j* to total output, reflecting each sector’s direct water use intensity in producing the sector’s products. Then, the complete water consumption matrix for each sector can be expressed as follows:

(19) $$W=\omega X=\omega {\left(I-A\right)}^{-1}Y$$

The direct water use coefficient reflects the direct water use intensity of each sector in the production of its own products, while the full water use coefficient examines the indirect relationship between each sector from the end-use perspective, which represents the increase in water use of the entire economic system when a sector produces a unit of product:

(20) $$\tilde{\omega }=\omega {\left(I-A\right)}^{-1}$$

##### Water Resource Shadow Price Model

By further expanding the water resources input-output analysis method, a water resources shadow price calculation model is constructed to solve for the shadow price of water resources in the receiver areas and to analyze the economic benefits of water transfer. The shadow price of water resources is the marginal contribution of the optimally allocated unit of water resources to socioeconomic development [65]. By analyzing the input-output situation of water resources in each sector in the receiver areas, finding the target optimization sector, maximizing the efficiency of water resource utilization as the goal, and determining the constraints based on the relationship between the output value of each sector and the total available water resources based on Equation (18), the final model for calculating the shadow price of water resources is established as follows:

(21) $$Z=\max\sum_{i=1}^{n}X_{i}$$

where *Z* denotes the pursuit of the target variable, i.e., the maximum total output. $X_{i}$ denotes the output of sector *i*. Finally, the product of the derived shadow price and the amount of water transfer used by the receiver areas is recorded as the economic benefit of water diversion, which lays the foundation for determining the ecological compensation standard.

##### Cost Analysis Model with the Willingness to Pay

According to the principle of “who benefits, who compensates”, the receiver areas, as the beneficiary areas of the cross-basin water diversion projects, should compensate the main headwater areas according to the corresponding ecological compensation standards if the water quality meets the national standards. This paper combined the willingness to pay cost analysis method to determine the ecological compensation standard of the receiver areas.

With the development of society and the continuous improvement of people’s living standards, people’s awareness of ecological values and willingness to pay for ecological compensation have also been changing [48,66]. To prevent large deviations across the calculation results of the ecological compensation standards and the willingness to pay by the receiver areas, the regional development coefficient is introduced to indirectly express the willingness to pay ecological compensation by the receiver areas; the calculation formula is as follows:

(22) $$L=\frac{1}{1+e^{-t}},t=\frac{1}{E_{n}-3}$$

where *L* denotes the regional development stage coefficient and $E_{n}$ denotes the Engel coefficient.

Combined with the ecological compensation payment willingness of the receiver areas, the product of the cost required to obtain the same amount of water resources without water diversion projects and the regional development stage coefficient are used as the ecological compensation standard. The receiver areas are not compensated separately for the part of the transferred water that exceeds their shortage. Therefore, the calculation formula for determining the ecological compensation standard by combining the cost analysis method of the willingness to pay is as follows:

(23) P=λ×L×Q×S×H

where *P* denotes the compensation standard and λ denotes the determination coefficient when water quality standards transfer to 1 and 0 otherwise. *Q* denotes the transfer of water, *S* denotes the shadow price of water resources, *H* denotes the water price standard, and this paper selected the cost of sewage treatment as the water price standard.

## 5. Results

### 5.1. Eco-Compensation Standard Based on the Total Cost Correction Model

#### 5.1.1. Direct Cost Accounting of Main Headwater Areas

The main headwater areas are the core of the entire water diversion project; the quality of the main headwater areas directly affects the water security and economic development of the receiver areas along the route [67]. Main headwater areas have sacrificed greatly to protect water sources by continuously adjusting their economic structures and development methods, controlling the total amount of pollutants discharged, and managing the ecological environment of the basin to ensure a long-term stable supply of clean water resources. Hence, it is necessary to fully evaluate the direct costs paid by the main headwater areas since the commissioning of the water diversion project.

MRP was officially opened for water transfer in December 2014, so the years from 2015 to 2020 were selected as the accounting years for the total cost. To evaluate the direct cost inputs of watershed ecological compensation of the main headwater areas (Hanzhong, Ankang, Shangluo, and Shiyan), a total of five direct expenditure indicators were selected with reference to previous studies [68,69]: hydrological monitoring costs, water pollution control costs, soil and water conservation costs, forestry construction costs, and watershed ecological construction costs.

**Figure 2:**
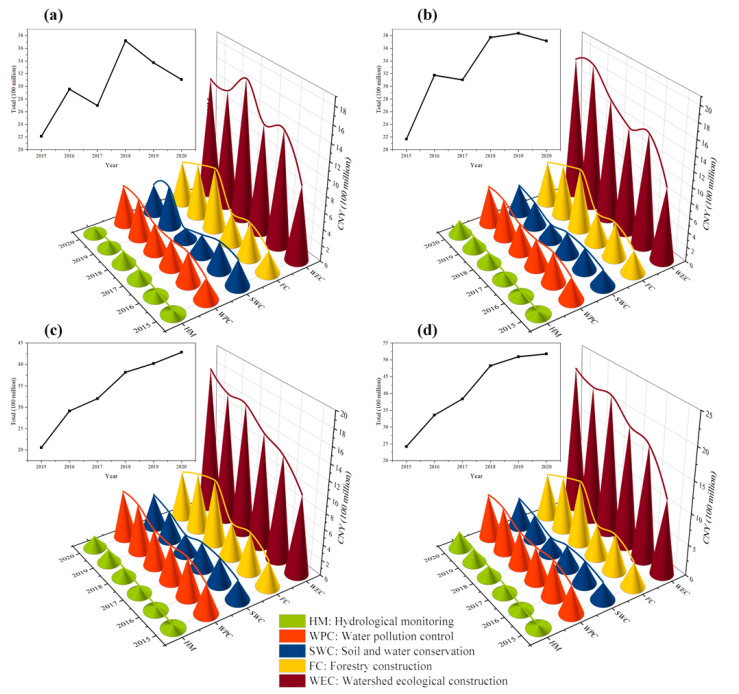
Direct input costs of (**a**) Hanzhong, (**b**) Ankang, (**c**) Shangluo, and (**d**) Shiyan among the main headwater areas from 2015 to 2020.

As shown in Figure 2, the overall direct input costs of the main headwater areas show a fluctuating upward trend. In six years, the accumulated direct input cost is CNY 82.835 billion and the calculated average annual expenditure is CNY 20.709 billion. From the administrative district, Shiyan City has the highest cumulative direct input cost of CNY 24.712 billion, which is probably because the Danjiangkou Reservoir, which is the main headwater area, is located in Shiyan City and the area of rainfall into the Danjiangkou Reservoir is 2.09 square kilometers, accounting for 88.1% of the city’s area, which is the core area for water source environmental protection of the water diversion project [70]. In addition, Shiyan’s per capita GDP is significantly higher than that of other municipalities in the main headwater areas and more funds are at its disposal for ecological construction. In terms of individual indicators, watershed ecological construction projects spend the most, with cumulative expenditures of CNY 40.84 billion in four municipalities, which accounts for 49.3% of total input costs. The main headwater areas are located in the upper reaches of the HJRB, one of the most resource-intensive areas in central and western China. Since 2012, the government has paid more attention to constructing ecological civilization based on the basin, with policies and funds tilted more toward this area [71].

#### 5.1.2. Opportunity Cost Accounting of Main Headwater Areas

To ensure that the water diversion project has a long-term benefit, it is necessary to ensure that the water diversion amount and quality meet the requirements of the water diversion project [72,73]. In this process, the main headwater areas not only need to invest a large amount of money to maintain the watershed environment but also to restrict or even shut down some enterprises, which to a certain extent limits socioeconomic development and reduces the residents’ qualities of life in the main headwater areas [74]. Therefore, it is necessary to assess the opportunity cost of the main headwater areas from industrial and agricultural points of view since the commissioning of the water diversion project.

##### Opportunity Cost of Producers in the Secondary Industry

To coordinate the basin-wide development situation, considering the interests of all parties and improving the environmental quality of the main headwater areas, the government of the main headwater areas usually introduces relevant policies. It implements a more stringent industrial access system [75]. On the one hand, some of the more environmentally harmful enterprises, especially high energy-consuming and highly polluting enterprises, will be relocated, banned, closed, forced to shut down, and other administrative measures are adopted for enterprises such as the ones involved in heavy metals, mining, metallurgy and textile industries; on the other hand, the government of the main headwater areas will implement a more stringent discharge permit system and set strict discharge standards and only enterprises that meet the standards will be permitted to continue with normal production.

**Figure 3:**
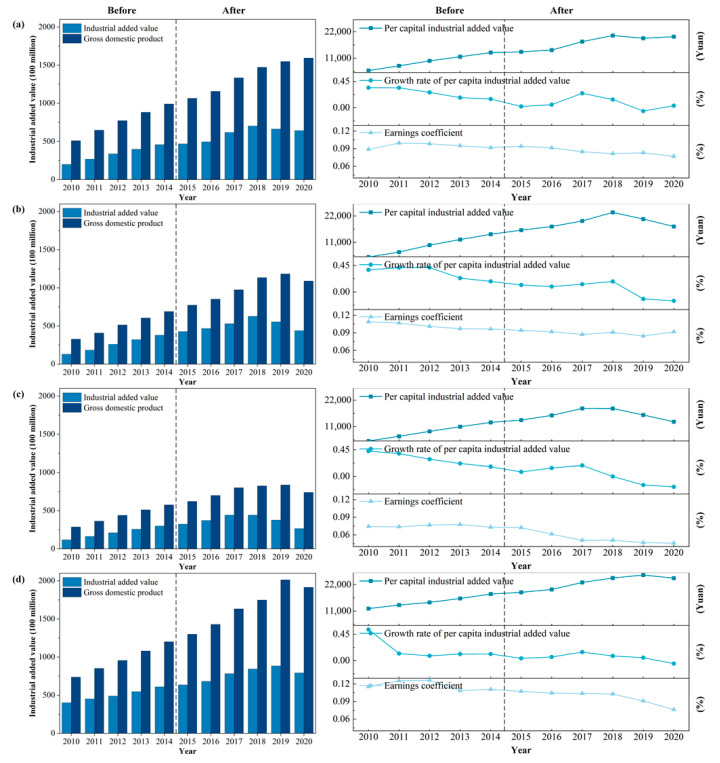
Changes in the main economic indicators of (**a**) Hanzhong, (**b**) Ankang, (**c**) Shangluo, and (**d**) Shiyan among the main headwater areas before and after the start of the middle route project from 2010 to 2020.

Figure 3 shows the changes in the main economic indicators of the government of the main headwater areas before and after the start of the water diversion project. The total GDP of the main headwater areas has shown an overall growth trend. However, the industrial value added per capita has grown slowly since 2014 (the project start-up year), with negative growth in individual years. Among them, the growth rate of industrial value added per capita in Hanzhong City decreased from 14.93% in 2014 to 2.19% in 2015, a decrease of more than 85.33%, which was most affected by the water diversion project. In addition, Ankang, Shangluo, and Shiyan’s drops were 33.70%, 53.54%, and 65.08%, respectively. It is worth noting that the growth rate of industrial value added per capita in Shangluo City was negative for the first time in 2018, being the earliest city in the main headwater areas to experience negative growth; thus, presumably, Shangluo City’s industrial economy may be more vulnerable compared with other cities in the main headwater areas.

The above observations show that the banning and restriction of the production activities of some industrial enterprises, with the year of the project start up as the dividing line, has greatly affected the industrial development of the main headwater areas. The ecological protection behavior of the main headwater areas has led to a large loss of opportunity cost for industrial producers, which will create the gap between the industrial development and income level of the main headwater areas as other cities continue to experience increased growth.

To quantify the industrial opportunity cost loss, Weinan, Yan’an, Tongchuan, and Huangshi, which are geographically close to the main headwater areas and have comparable levels of economic development, were selected as reference areas to calculate the opportunity cost loss of the main headwater areas, as shown in Table 3. The change in industrial opportunity cost losses in the main headwater areas from 2015 to 2020 is generally relatively flat. Shiyan has the largest opportunity cost loss of CNY 2.647 billion and Shangluo has the smallest loss of CNY 1.843 billion. In terms of the total volume, the largest losses were incurred in 2019, amounting to CNY 1.604 billion, with a cumulative six-year industrial opportunity cost loss of CNY 9.067 billion. This is a potential loss of revenue that cannot be ignored for the government of the main headwater area, which is already relatively slow in industrial development.

##### Opportunity Cost of Producers in the Primary Sector

The government of the main headwater areas is under heavy pressure to protect the environment and usually adopts strong administrative measures to intervene in agricultural production in the area. Agriculture can be roughly divided into plantation and nonplantation industries. In the plantation industry, the project of returning farmland to forest may be implemented, directly leading to a significant reduction in the area planted with cash crops such as medicinal herbs and tea [76]. Moreover, strict controls on agricultural fertilizers can also lead to varying degrees of decline in agricultural yields. On the non-planting side, the economic forestry transition to public welfare forestry projects will reduce farmers’ incomes from forestry. Adopting a stricter access system for the livestock industry would significantly reduce livestock farming enterprises. The high standard of discharge requirements for pollutants such as livestock sewage and manure increase the cost of additional expenditures for this type of enterprise. In addition, to improve the water quality condition, the government of the main headwater areas will change part of the fishery farming water area into natural wetlands and strictly prohibit artificial farming methods such as net box farming that use an excessive amount of drugs. These initiatives will undoubtedly lead to a decrease in fishery production and cause a large opportunity cost loss to agricultural producers.

**Figure 4:**
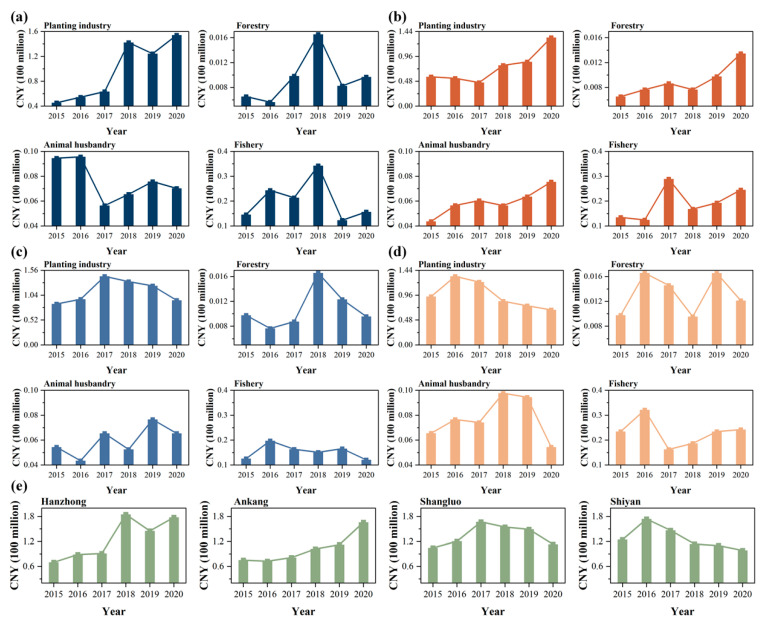
The opportunity cost of primary industry in (**a**) Hanzhong, (**b**) Ankang, (**c**) Shangluo, and (**d**) Shiyan among the main headwater areas from 2015 to 2020, (**e**) total opportunity cost changes of primary industry.

Figure 4 illustrates the loss of agricultural opportunity costs in the main headwater areas from 2015 to 2020, with a cumulative loss of CNY 2.941 billion. In terms of the industry type, the largest opportunity cost loss was in the plantation industry, which was CNY 2.283 billion, accounting for approximately 77.66% of the total agricultural opportunity cost. Forestry had the smallest opportunity cost loss of CNY 25 million, accounting for only 0.86% of the total agricultural opportunity cost. In terms of the administrative regions, Shangluo City had the largest total agricultural opportunity cost loss of CNY 809 million, followed by Shiyan with CNY 767 million, Hanzhong with CNY 757 million, and Ankang with CNY 608 million. The opportunity cost loss of agriculture in the main headwater areas after the start of the water diversion project comes from the plantation industry.

#### 5.1.3. Total Cost Accounting of Introducing Water Quantity and Water Quality Coefficient

##### Water Quantity Allocation Coefficient

The water quantity allocation and quality correction coefficients are introduced to make appropriate corrections to the direct costs and opportunity costs so that the final total cost amount can be measured. Among them, the water quantity allocation coefficient is determined by the water diversion amount and the total water supply in the HJRB. To continuously deliver sufficient water to the receiver areas, a series of water conservation construction projects are carried out in the main headwater areas to maintain water quantity and increase ecological benefits, including afforestation and forest nurturing measures.

Table 4 shows the water diversion amount and the total water supply in the HJRB from 2015 to 2020, indicating significant changes in annual water transfers. The receiver areas are affected by precipitation, storage, and other factors, resulting in annual changes in water demand; thus, there are differences in the annual water diversion amount and the overall trend is increasing year by year. Correspondingly, the water quantity allocation coefficient increases yearly with the increasing water diversion amount.

##### Water Quality Correction Coefficient

Water quality will also affect the benefits of water diversion projects; there is a need to introduce a water quality correction coefficient to further adjust the ecological compensation standards. In this paper, we select the water diversion project’s intake Tao Fork site. According to the China General Environmental Monitoring Station’s water quality automatic monitoring report of key cross-sections of the main national watershed, we select the hydrogen ion concentration index (pH), dissolved oxygen (DO), potassium permanganate salt index (COD_Mn_), and ammonia content in water (NH3-N) as the evaluation index of water quality correction. According to China’s current Environmental Quality Standards for Surface Water (GB3838-2002), resource quality is divided into five high and low categories, as shown in Table 5. According to the “Environmental Protection Guidelines for Centralized Drinking Water Sources” issued by the Ministry of Environmental Protection, when the water quality standard reaches Class III, it can be used as centralized drinking water. Therefore, this paper selects the multiyear average value of pollutant index monitoring data and calculates the water quality correction data according to the implementation standard of Class III water quality.

Table 6 shows the annual average values of water quality standards for Class III and monitoring data for pollutant indicators at the Tao Fork site. Since 2015, the section of the four major pollutant index data is to meet the Class III water quality standards, almost to meet the Class I water quality standards, which shows the main headwater areas protection results of the water quality of the project delivery are significant and the receiver areas are enjoying the water quality benefits. Consequently, the overall trend of the water quality correction coefficient is increasing yearly.

##### The Revised Total Cost

**Figure 5:**
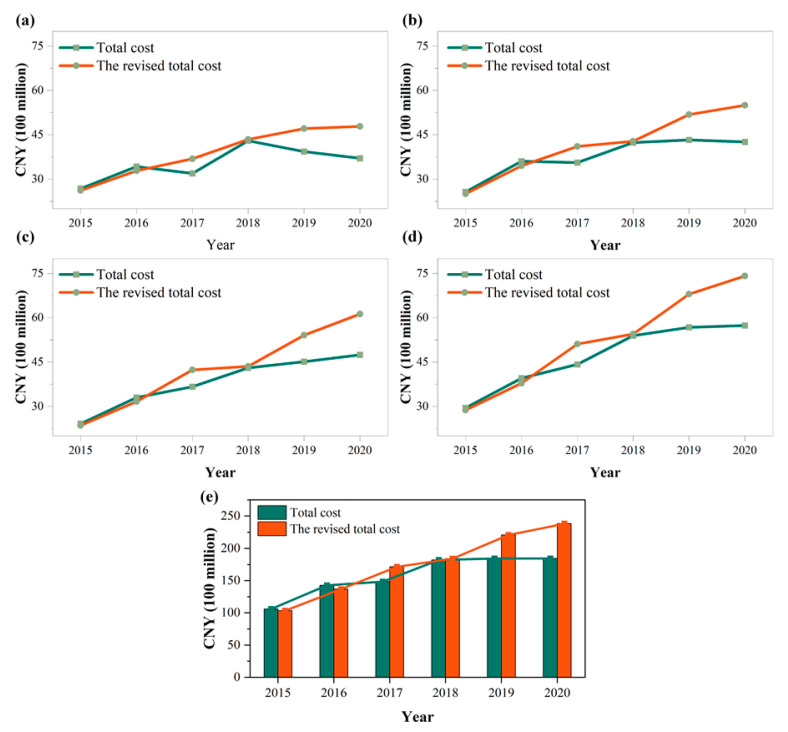
Changes in the total cost and revised total cost of (**a**) Hanzhong, (**b**) Ankang, (**c**) Shangluo, and (**d**) Shiyan among the main headwater areas from 2015 to 2020 and (**e**) all main headwater areas.

The total cost is calculated based on the direct cost and opportunity cost. The corrected total cost is calculated after introducing the water quantity allocation coefficient and the water quality correction coefficient, which are CNY 10.350, 13.676, 17.146, 18.426, 22.098, and 23.821 billion for the consecutive study years, respectively, and the six-year cumulative compensation value is CNY 105.516 billion. Its trend is shown in Figure 5. The increasing trend in total cost after correction is significantly higher than the total cost. At the municipal level, the total cost of each municipality in the main headwater areas is higher than the revised cost in 2015 and 2016, which is perhaps due to the overall low water transfer volume in the first two years after the start of the water diversion project, resulting in a smaller water quantity allocation coefficient. In addition, the total cost in 2018 was almost flat with the revised cost, and was CNY 18.225 and 18.426 billion, respectively; the reason is perhaps due to the increasing trend of the potassium permanganate salt index (COD_Mn_) and ammonia content in the water (NH3-N) index in 2018, the decreasing trend of the dissolved oxygen (DO) index, and the deterioration of water quality monitoring data at the cross-section. However, it still meets the Class I water quality standard. There is a slight decline compared with other years, resulting in a smaller water quality correction coefficient. In addition, the gap between the total cost and the revised cost in 2019 and 2020 became increasingly obvious, which is perhaps due to the increasing water diversion amount and the improved quality of water delivered, resulting in an increased water quantity allocation coefficient and an increased water quality correction coefficient.

### 5.2. Eco-Compensation Standard Based on the Water Resources Input-Output Model

#### 5.2.1. Shadow Price of Water Resources

The value of water resources has a theoretical basis for formulating reasonable water prices, which is of great significance in promoting the rational allocation of water resources and alleviating the contradiction between water supply and demand. [77,78]. The shadow price of water resources refers to the marginal contribution of unit water resources to economic and social development after optimal allocation, which is the basis for measuring the value of water resources. To calculate the economic benefits generated using water resources in the receiver areas, this paper calculates the 2015 water resource shadow prices based on the regional input-output analysis of the receiver areas of Henan (Nanyang, Pingdingshan, Xuchang, Zhengzhou, Jiaozuo, Xinxiang, Hebi, Anyang), Hebei (Handan, Xingtai, Shijiazhuang, Baoding), Beijing, and Tianjin in 2015. Considering the continuous socioeconomic development, the economic benefits per unit of water resources for all national economic sectors are increasing yearly. Therefore, the interannual variation in the shadow price of water resources is represented by the GDP growth rate of the receiver areas. The shadow price of water resources in other years is calculated to determine the economic benefits of water diversion in the receiver areas.

Table 7 shows the results of the total output of the receiver areas in 2015 under the optimal water allocation condition. By solving the paired problem of the water resources optimization model, the shadow prices of water resources in 2015 were calculated to be 4.73 CNY/m^3^, 3.81 CNY/m^3^, 3.23 CNY/m^3^, and 3.05 CNY/m^3^ in Henan, Hebei, Beijing, and Tianjin, respectively. A shadow price greater than zero indicates water scarcity and the greater the scarcity is, the greater the shadow price. The shadow price of local water resources in each receiver area is greater than zero, indicating a general scarcity of water resources throughout the receiver areas. The size of the shadow price shows that the local water resources in Henan and Hebei are relatively scarce and the conflict between water supply and demand is prominent. In contrast, the conflict between water supply and demand in Beijing and Tianjin is relatively moderate. The results reflect the marginal contribution of limited water resources to economic and social development under optimal allocation and can provide technical support for formulating scientific and reasonable water price standards and improving the water price system in the South-North water diversion receiver areas.

#### 5.2.2. Economic Benefits of Water Diversion

Currently, most countries in the world produce input-output tables only at intervals of several years [63]. The shadow price of water resources in 2015 was calculated based on the input-output table of the receiver areas in 2015. However, with the continuous economic development, the importance of water resources is bound to change and the economic benefits per unit of water resources for socioeconomic development will rise annually. Based on the GDP growth rate of the receiver areas from 2015 to 2020 and the water diversion amount, the shadow price of water resources from 2016 to 2020 is calculated. The product of its use and the water diversion amount in the corresponding year are used as the economic benefit obtained by the receiver areas through the water diversion project.

**Figure 6:**
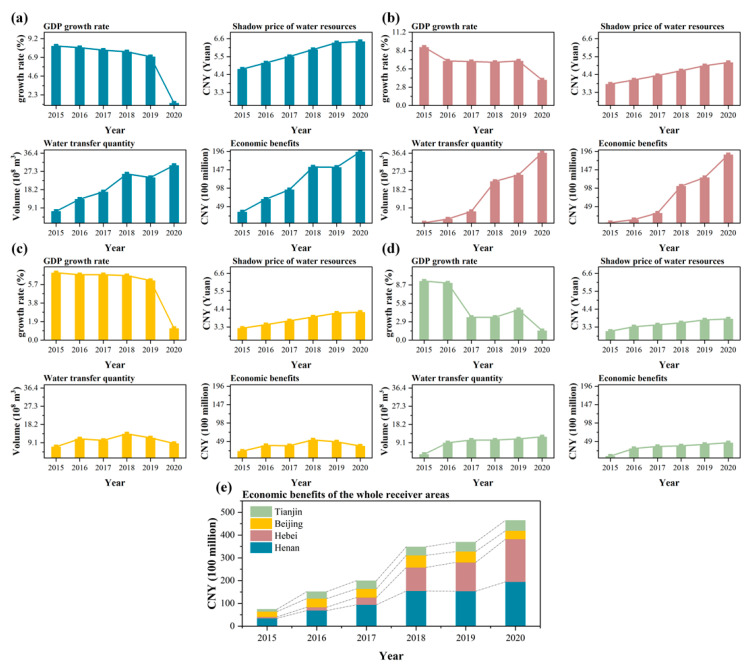
Economic benefits in (**a**) Henan, (**b**) Hebei, (**c**) Beijing, and (**d**) Tianjin in the receiver areas from 2015 to 2020 and (**e**) all receiver areas.

As seen from Figure 6, with the continuous socioeconomic development of the receiver areas, the shadow price of water resources is rising yearly. In addition, the economic benefits of the receiver areas are similar to the changing trend of the water diversion amount, with a total of CNY 160.866 billion of economic benefits obtained in six years. In terms of administrative regions, the most significant gain from the water diversion project is CNY 70.116 billion in the Henan Province, CNY 47.024 billion in the Hebei Province, CNY 23.674 billion in Beijing, and CNY 20.052 billion in Tianjin. The increase in water diversion amount yearly eases the contradiction of water supply and demand in the receiver areas and creates considerable economic benefits. The calculation and analysis of the economic benefits of water diversion provide an important basis for calculating the ecological compensation standards in water receiver areas.

#### 5.2.3. Eco-Compensation Standard with the Cost Analysis Method

The continuous development of the national economy depends on the support of resources and the progress of resource utilization technology. In recent years, water resource utilization in the receiver areas has gradually stabilized and national economic development mainly has relied on more efficient resource utilization [79]. Therefore, resource use efficiency growth can be considered as synchronized with the development of the national economy. Considering the economic development factor, the GDP growth rate of the receiver areas represents the inter-year change in the water resources shadow price. The compensation standard to be paid by the receiver areas is calculated based on the economic benefits gained by the receiver areas from the water diversion project, combined with the sewage treatment cost of the tap water supply.

**Figure 7:**
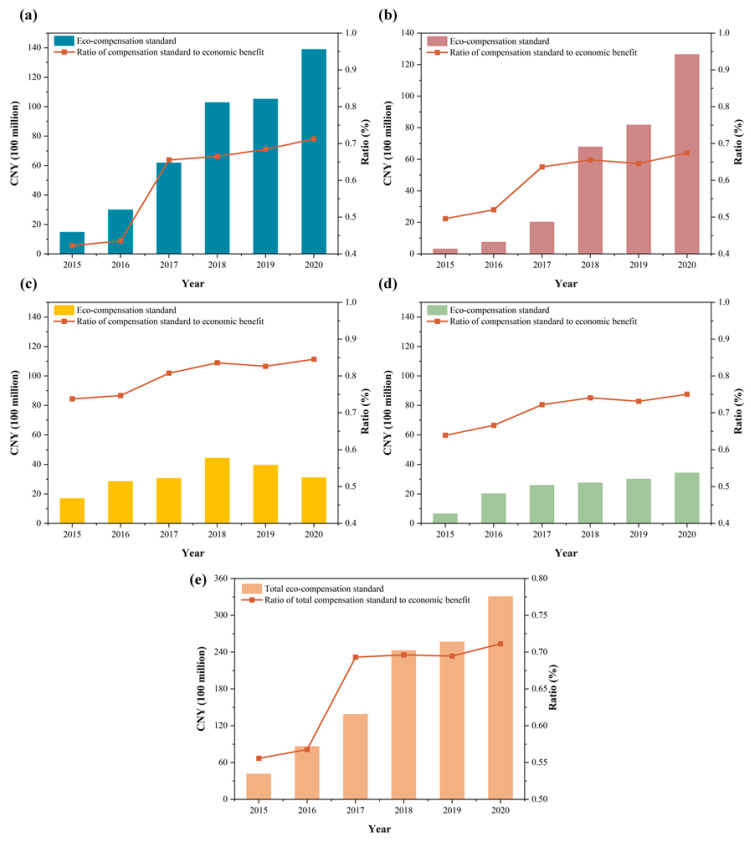
Changes in the eco-compensation standards of (**a**) Henan, (**b**) Hebei, (**c**) Beijing, and (**d**) Tianjin in the receiver areas from 2015 to 2020 and (**e**) all receiver areas.

As shown in Figure 7, the ecological compensation standard of each receiver area generally shows an increasing trend from 2015 to 2020, with only a slight decrease in the compensation amount from 2018 to 2020 in Beijing, which is perhaps due to the ecological compensation standard change with the annual water transfer, which is 1352, 115.3, and 874 million m^3^ from 2018 to 2020 in Beijing, showing a decreasing trend year by year. In addition, the ratio of compensation rates to economic benefits generally showed an increasing trend year by year, with a more pronounced increase from 2016 to 2017, which is perhaps attributed to the increase in sewerage costs for the following year at the end of 2016 in many parts of the receiver areas. From an administrative point of view, Henan should bear the highest amount of ecological compensation, followed by Hebei. At the same time, Beijing and Tianjin are relatively low; this result is positively correlated with the water distribution amount.

Overall, the ecological compensation standards based on the water resources input-output model for the receiver areas from 2015 to 2020 are CNY 41.38, 86.14, 13.858, 24.253, 25.670, and 33.070 billion, respectively, with a cumulative compensation standard of CNY 109.603 billion in six years. The changing trend is shown in Figure 7e.

## 6. Discussion

### 6.1. Comparison of Two Models in the Water Diversion Project

This paper uses two models to calculate the ecological compensation criteria for the world’s largest cross-basin water diversion project based on the perspective of the main headwater and receiver areas. The total cost correction model is based on the main headwater areas’ direct costs and opportunity costs as the compensation standard, focusing on the direct and potential costs of the main headwater areas to protect water resources [80]. It is worth noting that since cross-basin water diversion projects are different from other common basins, water quality and quantity are important factors that sustain project operation and determine project benefits [28,81]. Therefore, this paper introduces the water quantity allocation coefficient and water quality correction coefficient to optimize the model, causing the traditional total cost model to be more suitable for ecological compensation standard accounting of cross-basin water diversion projects. In addition, the water resources input-output model is based on the economic and environmental benefits of the receiver areas as the accounting basis for the compensation standard using the calculation of the economic benefits generated by the use of the transferred water resources in the receiver areas, combined with the input-output table and cost analysis method to calculate the ecological compensation standard of the receiver areas.

**Figure 8:**
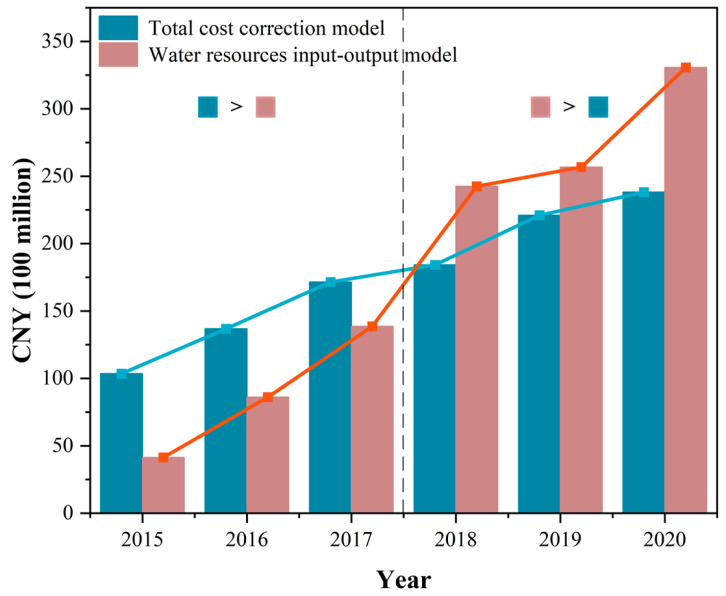
Comparison of the results of the total cost correction model and water resources input-output model.

China’s South-to-North Water Diversion Project is the world’s largest cross-basin water diversion project. It has greatly contributed to alleviating water conflicts in northern China and promoting coordinated regional development. Figure 8 shows the calculated results for both models. In terms of the changing trend, the ecological compensation standards calculated by both models showed an increasing trend year by year. The results of the total cost correction model show that the ecological compensation standards for the main headwater areas from 2015 to 2020 are CNY 10.350, 136.76, 17.146, 18.426, 22.098, and 23.821 billion, respectively, and the cumulative compensation value over the six years is CNY 105.516 billion, with a relatively flat annual growth trend. The water resources input-output model results show that the ecological compensation standard of the receiver areas in six years was CNY 4.138, 8.614, 13.858, 24.253, 25.670, and 33.070 billion and the cumulative compensation value was CNY 109.603 billion, with rapid annual growth.

It is noteworthy that before 2018 the ecological compensation standard calculated by the total cost correction model was significantly higher than that of the water resources input-output model, while the opposite is shown after 2018. While the total cost correction model is influenced by the input cost of the main headwater areas, the water resources input-output model focuses on the economic benefits that the receiver areas obtain from the water resources. At the beginning of the water diversion project operation, the water resources transferred to the receiver areas were relatively small and the economic benefits generated were relatively small. However, the direct and indirect costs to the main headwater areas when constructing the water diversion project are enormous. After 2018, the water diversion amount increased each year and the increase started to accelerate each year, which alleviated the water shortage in the receiver areas. The water diversion project’s economic benefits have become more obvious and the compensation standard calculated by the water resources input-output model has increased significantly.

### 6.2. Theoretical Contributions

In previous studies, scholars have conducted a lot of research on the ecological compensation standards based on the perspective of main headwater or receiver areas [38,82,83]. However, few scholars have measured the compensation standards from dual perspectives. Cost-based compensation is the minimum compensation for the implementation of watershed ecological protection, but it is difficult to reflect the equity and often provides insufficient incentives for ecological protectors. Water resources value-based compensation may lead to over-compensation, heavy financial burden, and funds used inefficiently [3]. On the other hand, in the previous literature, the evaluation method system of water resources value is not perfect and the accounting scope and indicators of cost measurement are not clear enough, which leads to a large difference in ecological compensation standards [4].

The main contribution of this paper is to modify two models to calculate the ecological compensation criteria for cross-basin water diversion projects from the perspective of the main headwater areas and the receiver areas. This paper refers to the practice of most scholars. It considers both direct and indirect environmental protection costs, based on which water quality and quantity correction coefficients are introduced to more accurately account for ecological compensation standards from the perspective of main headwater areas and better reflect fairness. Moreover, since the water resources value is difficult to determine and the water resources trading market in China is not yet mature, this paper uses the water resources input-output method to measure the economic benefits obtained by the water diversion project in the receiver areas, which reasonably avoids the drawbacks of the water resources value method [11].

### 6.3. Policy Recommendations

To promote the stable development of MRP of the SNWDP main headwater areas and receiver areas and to advance the stable implementation of ecological compensation in the watershed, the following policy recommendations are proposed based on the findings of this paper.

- Expand the funding channels for watershed ecological compensation. While the government strengthens financial vertical transfer compensation, it mobilizes various resources to support watershed ecological compensation as much as possible, actively expands multidimensional and multilevel diversified financing channels, and transforms “blood transfusion” compensation into “blood-making” compensation.
- Actively develop a more flexible ecological compensation method. Based on the principle of “who benefits, who compensates” to establish direct counterpart compensation between the receiver areas and the main headwater areas, the receiver areas directly decide the compensation standard of the reservoir area according to the annual water quality and quantity of the main headwater areas.
- Gradually establish a market for ecological services in main headwater areas, explore market compensation mechanisms for main headwater areas, and promote the conversion of government compensation to market compensation, thereby transferring a larger share of ecological values and economical construction costs to the market and reducing government financial pressure.

### 6.4. Limitations and Future Work

The total cost correction model integrates the opportunity cost method and cost analysis method, introduces water quality and water quantity correction factors, and mainly calculates the direct and indirect costs of the main headwater areas. The advantage is that it can quantitatively assess the economic costs paid by the main headwater areas for foregoing development opportunities and carrying out watershed ecological protection to protect the ecological environment of water sources. The calculation process is simple and intuitive to understand, but the calculation involves more cost items and the data collection is more difficult. More importantly, the reality is that there is uncertainty with respect to the cost of spending in the main headwater areas and the accuracy of the data used is limited. In addition, it is unfair to assume that the difference in per capita income levels is solely due to water conservation.

The water resources input-output model combines the value of the water resources method, shadow price method, input-output analysis, and cost analysis method and mainly calculates the economic benefits obtained by using the water resources in the receiver areas. Among them, the value of water resources is influenced by natural factors (abundance and quality of water resources, development conditions, characteristics of water resources, etc.) and economic factors (industrial structure, scale, water use efficiency, GNP, etc.) and social factors (technology, population, policies, cultural and historical background, etc.), while the results of this paper are obtained based on the current year’s water resources supply and demand situation in the receiver areas, the current status of water conservancy projects, price levels, and industry water use structure, which are somewhat time-sensitive.

In future work, the advantages of the two models can be combined. The difference between the ecological benefit of the receiver areas and the ecological cost of the main headwater areas can be used as the ecological compensation standard. The total cost correction model can be combined with the water resources input-output model to construct a basin ecological compensation standard econometric model based on the perspective of regional development imbalance to calculate a more accurate and scientific basin ecological compensation standard.

In addition, watershed ecological compensation is not a short-term process in terms of time scale, as it has a more extended period. In a long-term strategy, there is no guarantee that factors such as land use, vegetation cover, river runoff, socioeconomics, and the value of natural products will remain constant. Therefore, to adapt to the temporal and spatial changes in the indicators in the study area, a dynamic watershed ecological compensation accounting system needs to be constructed in the future.

## 7. Conclusions

This paper compares the differences between the total cost correction model and the water resources input-output model applied in water diversion projects from the perspective of the main headwater and receiver areas. The total cost correction model based on the main headwater areas perspective focuses on the cost paid by the main headwater areas, which is affected by the ecological construction expenditure and the industrial opportunity cost loss in the basin. In contrast, the water resource input-output model based on the receiver area perspective focuses more on the change in the project water diversion amount. The ecological compensation standard of MRP will increase yearly in the future. However, due to the gradual stabilization of ecological construction expenditure in the main headwater areas and the yearly increase in the project water diversion amount, the ecological compensation standard calculated based on the water resources input-output model will be larger than the total cost correction model. The findings of this paper provide a methodological and data reference for the ecological compensation mechanism of realistic cross-basin water diversion projects, which is conducive to enhancing the water transfer benefits of water diversion projects and promoting the synergistic development of the main headwater areas and receiver areas.

The construction of the water diversion project has caused an imbalance in the regional development of the main headwater areas and the receiver areas. Based on the rational division of labor and cooperation, the main headwater areas are mainly responsible for producing ecological products (providing high-quality water resources). In contrast, the receiver areas are mainly responsible for producing material products (using water resources), which leads to the increasingly prominent contradiction between ecological environmental protection and economic development in the basin. Establishing a watershed ecological compensation mechanism in the basin is urgent to realize the coordinated development of the ecology and economy. Establishing a perfect watershed ecological compensation system is an effective way to solve the externalities of ecological products in water sources and an important path to realize the coordinated development of main headwater areas and receiver areas. A clear ecological compensation standard is one of the core issues.

However, watershed ecological compensation is a systematic and complex multidisciplinary research field. This paper mainly studied the ecological compensation standards of water diversion projects with different models. However, it did not cover achieving compensation standards, cost-effectiveness, social welfare and equity, etc. These issues still need further in-depth research.

## Figures and Tables

**Table 1:** Advantages and disadvantages of different watershed ecological compensation standard accounting methods.

Method	Advantages	Disadvantages
Willingness-to-pay method	It is more frequently used internationally, taking into account the beneficiary’s willingness to pay and the service provider’s willingness to be reimbursed.	Highly subjective, incomplete representation of the functional value of the ecosystem.
Total cost accounting method	The results of the compensation standard are easy to accept and take full account of the cost paid by the service provider.	Its results usually take into account only the costs and have no incentive for the service providers.
Ecosystem service value method	The value of the entire ecosystem of the watershed is fully reflected.	Different calculation metrics lead to highly differentiated results, usually greater than the beneficiary’s willingness to pay.
Water resources value method	The calculation process is simplified to focus on water quantity and quality.	The calculation method is immature and does not consider other values of water ecology.
Water quality and quantity protection target accounting method	The basis for compensation standard accounting is adequate and is used in practice by many regions.	Large differences in the economic development of beneficiaries and service providers lead to a flawed compensation process.

**Table 2:** Types and indicator explanation of direct cost.

Type	Indicators	Indicator Explanation
Ecological environmental protection and construction	Forestry construction (FC)	Mountain reforestation project, the project of returning farmland to forest, public welfare forest construction, the subsidies of returning farmland to forest, forestry resources protection, and forest pest control.
	Soil and water conservation (SWC)	Soil erosion control and key soil erosion demonstration areas project construction and operation.
	Watershed ecological construction (WEC)	Nature reserve construction, ecological restoration projects, watershed environmental protection science and technology costs, and comprehensive environmental improvement.
Integrated pollutant management	Hydrological monitoring (HM)	Water quality and water quantity monitoring station construction, monitoring instrument acquisition, maintenance, and operation costs.
	Water pollution control (WPC)	Wastewater treatment facility construction, rural biogas facility construction, and change in agricultural farming methods costs.

**Table 3:** The opportunity cost of secondary industry producers in the main headwater areas from 2015 to 2020 (100 million).

	2015	2016	2017	2018	2019	2020	Total
Hanzhong	3.97	3.82	4.04	3.92	4.13	4.18	24.07
Ankang	3.24	3.58	3.72	3.63	3.79	3.76	21.71
Shangluo	2.54	2.69	2.96	3.35	3.42	3.46	18.43
Shiyan	4.04	4.23	4.36	4.52	4.69	4.63	26.47
Total	13.78	14.32	15.09	15.42	16.04	16.02	90.67

**Table 4:** Water quantity allocation coefficient of the main headwater areas from 2015 to 2020.

Year	Water Transfer Quantity of Middle Route Project (10^8^ m^3^)	Total Water Supply of the HJRB (10^8^ m^3^)	Water Quantity Allocation Coefficient
2015	20.2	560	1.036
2016	38.3	557	1.069
2017	44.92	572	1.079
2018	57.84	580	1.100
2019	69.16	575	1.120
2020	86.22	572	1.151

**Table 5:** Limits of various items of surface water quality (mg/L).

Index	Class I	Class II	Class III	Class IV	Class V
pH	-	-	6–9	-	-
DO	≥7.5	7.5–6	6–5	5–3	3–2
CODMn	≤2	2–4	4–6	6–10	10–15
NH3-N	≤0.15	0.15–0.5	0.5–1.0	1.0–1.5	1.5–2.0

**Table 6:** Water quality correction coefficient of the main headwater areas from 2015 to 2020.

Year	pH	DO (mg/L)	COD_Mn_ (mg/L)	NH_3_-N (mg/L)	Water Quality Correction Coefficient
Class III	9.00	5.00	6.00	1.00	-
2015	8.02	8.13	1.28	0.12	1.012
2016	7.71	8.03	1.87	0.07	1.028
2017	7.73	7.91	1.52	0.04	1.254
2018	7.70	7.89	1.43	0.09	1.123
2019	8.07	8.35	1.19	0.05	1.362
2020	7.81	8.58	1.33	0.03	1.521

**Table 7:** Optimization results for the total sectoral output of receiver areas in 2015.

Province	City	Total Output (100 Million)		Total Water Consumption (10^8^ m^3^)	Unit Water Resources Output (CNY)		Differential Value (CNY)	Shadow Price (CNY)
		Original	Corrected		Original	Corrected		
Henan	Nanyang	2522.32	2578.64	16.2300	155.41	158.88	3.47	4.73
	Pingdingshan	1335.40	1393.80	9.4132	141.86	148.07	6.20	
	Xuchang	2170.60	2264.70	8.0700	268.97	280.63	11.66	
	Zhengzhou	7345.40	7402.70	16.7325	438.99	442.41	3.42	
	Jiaozuo	1960.42	2019.81	27.8341	70.43	72.57	2.13	
	Xinxiang	2007.14	2069.89	15.6418	128.32	132.33	4.01	
	Hebi	717.25	736.17	4.5700	156.95	161.09	4.14	
	Anyang	1657.94	1729.02	11.1025	149.33	155.73	6.40	
Hebei	Handan	3145.43	3213.24	14.1200	222.76	227.57	4.80	3.81
	Xingtai	1764.73	1852.21	16.8502	104.73	109.92	5.19	
	Shijiazhuang	5440.60	5531.53	29.4686	184.62	187.71	3.09	
	Baoding	3000.34	3091.28	27.8000	107.93	111.20	3.27	
Beijing		24,779.10	24,902.35	38.2000	648.67	651.89	3.23	3.23
Tianjin		10,879.51	10,961.24	26.7720	406.38	409.43	3.05	3.05

## Data Availability

The data presented in this study are openly available in National Bureau of Statistics of China at http://www.stats.gov.cn/ (accessed on 23 October 2022).
